# Sleep Deprivation, Immune Suppression and SARS-CoV-2 Infection

**DOI:** 10.3390/ijerph19020904

**Published:** 2022-01-14

**Authors:** Beatrice Ragnoli, Patrizia Pochetti, Patrizia Pignatti, Mariangela Barbieri, Lucrezia Mondini, Luca Ruggero, Liliana Trotta, Paolo Montuschi, Mario Malerba

**Affiliations:** 1 Respiratory Unit, S. Andrea Hospital, 13100 Vercelli, Italy; beatrice.ragnoli@aslvc.piemonte.it (B.R.); patrizia.pochetti@aslvc.piemonte.it (P.P.); mariangela.barbieri@aslvc.piemonte.it (M.B.); lucrezia.mondini@aslvc.piemonte.it (L.M.); luca.ruggero@aslvc.piemonte.it (L.R.); liliana.trotta@aslvc.piemonte.it (L.T.); 2 Allergy and Immunology Unit, Istituti Clinici Scientifici Maugeri IRCCS Pavia, 27100 Pavia, Italy; patrizia.pignatti@icsmaugeri.it; 3 Pharmacology Department, Faculty of Medicine, Catholic University of the Sacred Heart, 20123 Milan, Italy; p.montuschi@imperial.ac.uk; 4 Faculty of Medicine, National Hearth and Lung Institute, Imperial College of Science Technology and Medicine, Airways Disease Section, London SW7 2BX, UK; 5 Department of Traslational Medicine, University of Eastern Piedmont, 28100 Novara, Italy

**Keywords:** sleep deprivation, immune system suppression, SARS-CoV-2 infection

## Abstract

Sleep health and its adaptation to individual and environmental factors are crucial to promote physical and mental well-being across animal species. In recent years, increasing evidence has been reported regarding the relationship between sleep and the immune system and how sleep disturbances may perturb the delicate balance with severe repercussions on health outcomes. For instance, experimental sleep deprivation studies in vivo have reported several major detrimental effects on immune health, including induced failure of host defense in rats and increased risk for metabolic syndrome (MetS) and immune suppression in humans. In addition, two novel risk factors for dysregulated metabolic physiology have recently been identified: sleep disruption and circadian misalignment. In light of these recent findings about the interplay between sleep and the immune system, in this review, we focus on the relationship between sleep deprivation and immunity against viruses, with a special interest in SARS-CoV-2 infection.

## 1. Introduction

The need of sleep in all animal species suggests that sleep plays a pivotal role in preserving vital functions. Over the past decades, many studies have revealed the strict relationship between sleep and physical or mental well-being. In particular, sleep and the immune system appear to be bidirectionally linked, especially during the body’s defense against disease [1]. For example, it is well established how the host immune response to external pathogens, such as viruses, can be negatively influenced by deprivation of sleep [2]. Conversely, a regular sleep routine boosts the immune system, ensuring appropriate and effective immune responses. In this regard, some authors have identified different effects of total sleep deprivation (TSD) in rats [3,4,5], in particular, a TSD-induced defect of host defense [6,7,8,9,10]. In previous studies, the effects of sleep deprivation were demonstrated also in human subjects including enhancement of evoked potentials, heightening of fantasy in fantasy-impoverished subjects and alleviation of endogenous depression. Sleep has also been involved in the plastic cerebral transformations during the learning and memory processes [11,12,13]. More recent studies highlighted the role of sleep in maintaining metabolic homeostasis either in animal models than in humans and a possible correlation between sleep deprivation and the onset of a metabolic syndrome (MetS) as a consequence [14]. This complex network may furthermore be related with changes in immune system that may interfere with a physiological response increasing the susceptibility to viral infections. The following sections provide an overview on published evidence about the interplay between sleep deprivation and immune responses in rats and humans, the two most heavily studied in vivo models.

## 2. Sleep Deprivation Effects in Rat Models

Some authors reported several major effects of sleep deprivation (SDES) arising in rats after TSD or paradoxical sleep deprivation (PSD) through the disk-over-water (DOW) method [3]. These effects listed in Table 1 were subsequently confirmed by other groups [4,5] (Table 1).

Altogether, these studies demonstrated that TSD and PSD inevitably lead to a sleep-related syndrome with harmful effects on psychophysical health. In particular, sleep-deprived rats tend to show specific characteristics of progressive energy burn, typical skin lesions, thermoregulatory unbalance, leading to death, features never described in stressed rats [3,4].

The past four decades have witnessed a major paradigm shift in the study of the effect of sleep disturbances on the host immune response to invading pathogens [4]. Early studies about alteration of immune functions in TSD and PSD rats, assessing the amount of splenocytes, mitogen-induced lymphocyte proliferation test, and in vitro and in vivo plaque formation in response to various antigens, did not report significant differences from control group, suggesting that sleep deprivation does not lead to immune suppression [6]. By contrast, Everson isolated opportunistic and facultative anaerobe microbes in the blood of 5/6 near-terminal TSD rats, but none in controls, indicating, for the first time, an association between TSD and impaired immune response [7]. Everson’s results were subsequently confirmed [8,9] even though in vivo studies have produced conflicting results [10] (Table 2).

Data supporting that the gut is one of the primary sites of immune system dysregulation came from experiments on TSD rats orally treated with a mix of broad-spectrum antibiotics, where no bacteria could be found in the gut, blood, liver, kidneys and mesenteric nodes. The fact that these antibiotic-treated TSD rats continued experiencing a body temperature decline, a profound catabolic state manifested by high food intake and weight loss, and died “on schedule” in the absence of systemic bacterial infection supports the notion that an impaired immune response, caused by TSD, may play a decisive role in rat death [8]. However, taking together published data on this matter, we do not have a clear picture of how specific the effects of TSD may be in these animals. Indeed, sleep is so physiological in mammals that effects of its deprivation could be quite similar since produced by similar pathways [3,4]. Moreover, Sun Q et al. showed the effects of sleep deprivation on different signaling pathways, in particular, reduced expression of insulin receptor substrate (IRS)/phosphoinositide 3-kinase (PI3K)/AKT and the mammalian target of rapamycin (mTOR) as well as FoxO1 signaling pathways. Another important axis, the one regulated by the long isoform of the leptin receptor (LepRb)-mediated JAK2/STAT3 resulted attenuated by sleep deprivation. Young rats sleep deprived also experienced an alteration of the genes involved in the transcriptional feedback loop regulated by CLOCK and BMAL1 proteins. All these events confirm that alteration of sleep length causes dysregulation of several pathways in the hypothalamus with consequences in the regulation of hunger and energy consumption [15].

## 3. Sleep Deprivation Effects in Humans

This section may be divided by subheadings. It should provide a concise and precise description of the experimental results, their interpretation, as well as the experimental conclusions that can be drawn. Many causes are responsible for loss or dysregulation of sleep and usually a multifactorial origin is recognized. Among the most common reported causes of sleep loss there are respiratory disorders like apnea, but also other neurological and psychiatric conditions such as insomnia, parasomnias, mood disturbances, restless leg syndrome, and psychosis. It has been shown that the sleep architecture may change with age; deep sleep (characterized by delta-waves) decreases while the proportion of lighter sleep increases; this may conduct to increased sleep dysregulation [16]. Sleep disorders are widely distributed, as reported by different studies. Sleep loss affects more than 50 million people in America [17]. Léger D et al. showed that in 20% of the young adults analyzed (range 25–45 years old), sleep was reduced by ninety minutes as compared to what is needed for wellbeing [18]. Other authors reported in the last thirty years a consistent decrease in the duration of sleep coming up to about 18 min for night [19,20]. The accelerated rhythms of our society, which keep us ever connected, for work or pleasure, using computers, mobile phones and other devices until late at night, will likely determine an increase of sleep disorders. The effects of sleep deprivation on health outcomes have also been heavily studied in humans. From these studies, it seems that the most frequently observed effect in both TSD rats and humans is increased hunger [11,12,13]. Moreover, a 72-h TSD in humans significantly increased urea excretion, as well as plasma urea nitrogen increased in TSD and PSD rats in TSD and PSD rats [21]. Increased appetite in PSD subjects was also reported by Dement and Sampson [22,23]. However, psychological effects of sleep loss are not as quickly evident in humans as in rats. If 13.6 h in rats vs. 8.0 h in humans are considered the mean sleep need, the effects of TSD should onset 1.7 time faster in rats than in humans [3]. Studies on PSD in humans did not show evident damaging symptoms but they were relatively short (range 1–16 nights) [24]. The first report on long-time PSD results was published by Wyatt et al., who chronically administered phenelzine, a monoamine oxidase inhibitor (MAOI), in seven narcoleptic subjects, inducing severe and prolonged PS loss. Based on periodic recordings, in two subjects, PS almost disappeared for more than a year, with only mild symptoms reported as side effects of the drug mainly increased weight for excessive eating [25]. It should, however, be pointed out that in the aforementioned studies, cataplexy occurred when there were present either short intervals of PS or PS like moments [3]. Further studies showed how PSD could affect cerebral activity. These effects included enhancement of evoked potentials, boosting of fantasy in subjects with poor imagination and relief of endogenous depression [26]. Sleep has also been involved in neuroplasticity and in the learning and memory processes. Many published data confirmed that sleep is implicated in the fixation of short-term memory. Subjects who were sleep-deprived during post-training nights showed virtually no performance improvement on the following days, whereas subjects allowed to sleep immediately after training displayed a significant performance enhancement [27].

Among risk factors for human healthiness, sleep deprivation is one of the most changeable.

The American Heart Association and the Centre for disease Control and Prevention (CDC) claim that a sleep period of seven hours/night is necessary for favouring well-being and decreased probability of diseases [28,29]. The effects of inadequate sleep are dangerous.

In fact, morbidity and mortality connected to cardiovascular diseases (CVD) are dangerously increased by sleep disorders [30,31]. Everyday rhythm is present in cardiac, smooth muscle, and endothelial cells, modulating heart rate, blood pressure, and endothelial activity, [32] all functions with daily variation. Endothelial dysfunction and platelet activation are the trigger of atherosclerosis processes, already present before clinical presentation of CVD [33,34,35]; and for this reason, a significant target for evaluating the initial effects of sleep deprivation on the cardiovascular system. Moreover, endothelial activity evaluated in coronary and peripheral arteries reveals following CVD episodes (i.e., myocardial infarction and stroke) and death [36,37]. This is particularly true in subjects with chronic respiratory diseases such as COPD during stability phases [38,39] and during exacerbations [40]. Sleep deprivation may favour the onset and progression of age-related endothelial dysfunction and through this process favour pathogenesis and development of CVD [41]. Consequently, understanding the involvement of sleep in maintaining endothelial function may be useful to reach a healthy vascular aging.

The impacts of different hormones levels have been found to have a pivotal role in sleep deprivation. In fact, a chronic deprivation of sleep seems to be associated with increased cortisol levels and conversely with decreased levels of testosterone. Testosterone is known to intensify the function of the main blocker of neurotransmissions (gamma-aminobutyric acid—GABA) and of serotonin, involved in the regulation stabilization of mood and depression. The reduced testosterone levels could be involved in the relationship between depression and anxiety. Furthermore, elevated serum cortisol levels are associated with depression, anxiety, hypertension, obesity, and type II diabetes. Chronic sleep deprivation is associated with high inflammatory mediators, which are elevated in both comorbid conditions and mental illnesses.

The subjective experience of sleep loss can be distressing and conduct to increased cortisol levels, which can increase blood sugar, blood pressure and cravings for carbohydrates causing weight gain and consequently either medical and psychiatric problems. For the aforementioned reasons sleep loss can have painful and detrimental effects depending on subjects and can worsen pre-existing conditions [42].

## 4. Sleep and Metabolic Homeostasis

Sleep has a central role in preserving vital functions, as demonstrated by its conservation in all animal species [43,44]. Sleep is controlled by the genes and the neural network. However, these determinants interact with environment factors to influence sleep habit. Data have been published on the pivotal role played by cortical potassium channels in the overall control of sleep time [45]. Recently, experiments performed in Drosophila used as model for sleep, showed that specific voltage-gated potassium channels (“Shaker”) regulate sleep through GABAergic neurons sensitive to temperature [46]. Furthermore, it has been shown that potassium channels exert a complicated and time dependent impact on the cortical sleep EEG, in addition to a surprising control of the relationship between transcriptomic changes and EEG capacity [47]. Potassium channel modulators have also been demonstrated for potential therapeutic use in asthma and chronic obstructive pulmonary diseases in humans [48] confirming their enigmatic and fascinating role.

Xie et al. reported that sleep plays a pivotal role in determining homeostasis of metabolic functions [49]. Using real-time assessment of tetramethylammonium diffusion and two-photon imaging in live mice, these authors showed that natural sleep or anesthesia resulted in a 60% increase in interstitial space, followed by an important increase in the convective exchange of cerebrospinal with interstitial fluid. In addition, the convective fluxes of interstitial fluid increased the clearance of beta-amyloid during sleep. Thus, according to the authors, the restful function of sleep might be the result of the enhanced elimination of conceivably neurotoxic waste products accumulated in the central nervous system (CNS) during the waking state. Congruently, epidemiological data supported a correlation between shortened sleep and decreased lifespan, suggesting that less-than-optimal sleep duration might be deleterious to longevity in humans [50]. Finally, a recent review on sleep control and waking state has further confirmed that sleep disruption interferes with physiological restorative functions of either non-rapid eye movement (NREM) and rapid eye movement (REM) sleep, resulting in breathing dysregulation, altered cardiovascular function, changes in emotional reactivity and cognitive impairments in attention, memory and decision-making, with serious consequences in activities of daily living (ADL). Even though increased frequency of workplace or car accidents are obvious correlates, sleep disruption may also negatively influence the life of an individual in more subtle ways interfering with many activities: wakefulness, attention, working memory, conscious awareness, emotional regulation and/or memory consolidation in the adult, REM sleep and muscle control [51].

## 5. Sleep Deprivation and MetS

MetS diagnosis includes the presence of different symptoms such as visceral obesity, high blood pressure, a low blood concentration of high-density lipoproteins, increased triglycerides and insulin resistance/glucose intolerance, which is directly correlated with an increased probability to develop cardiovascular diseases. Indeed, three or more of these five conditions are enough to diagnose MetS [52]. Given that a function of sleep is the regulation of energy metabolism across animal species, the association of MetS with sleep in humans is not surprising. Indeed, an imbalance in circadian regulation of sleep leads to MetS in animals [53]. Moreover, homozygous clock mutant mice with reduced diurnal rhythms of feeding overeat, get fatter and display increased blood metabolic hormones, such as insulin and leptin and pathologic variation in hypothalamic hormones, which regulate food intake [54]. Intriguingly, similar metabolic defects were observed in knockout mice lacking circadian regulatory genes [55]. Even though epidemiological studies on humans suggesting that sleep alteration is related to MetS symptoms [56,57], a cause-effect link between these two conditions in subjects suffering from MetS it is still to be demonstrated. However, restoration of adequate sleep time and quality in sleep-deprived patients may be useful for reducing glucose and energy metabolism alterations. Conversely, increased oxidative stress in night shift workers enhances the risk of MetS [58]. Consistently, short sleep duration increased the incidence of MetS manifestations, twice in subjects sleeping below 6 h/night [59]. The influence of sleep and of its alterations on the hypothalamic–pituitary–adrenal (HPA) axis activity is widely known. Acute and chronic sleep deprivation may change the 24-h plasma concentration of cortisol, thyrotropin, prolactin and growth hormone. Furthermore, insomnia may change the cortisol rhythm [60]. Sleep deprivation may also alter insulin secretion after glucose intake, but sleep recovery restores physiological values [61]. Increase in body weight, obesity and diabetes have all achieved worrying degrees in the industrialized countries. Usual risks factors, like binge eating, inadequate nutritional options and sedentary habit, cannot completely explain the increased prevalence of metabolic diseases. A recent review points to sleep disruption and circadian misalignment as two novel risk factors of dysregulated metabolism [62]. In particular, the authors reported excessive caloric consumption as a consequence of decreased sleep, eating not when it is physiologically required, reduced energy consumption, when initiation of waking state and sleep are not compatible with physiological biorhythms, and altered glucose assimilation during brief sleep and circadian rhythm imbalance. Results of a recent meta-analysis evaluating data of 21 studies, showed that reduced duration of sleep is associated with higher concentrations of ghrelin, an orexigenic hormone favoring the gastrointestinal peristalsis and lowering insulin production [63]. Furthermore, in the subgroup of experimental studies analyzed, subjects who experienced reduced sleeping, had higher leptin levels, suggesting a dysregulation of hormones involved in the energy balance [63].

Thus, besides the usual risk factors like unhealthy eating and reduced physical activity, sleep disorders and altered circadian clock might represent treatable traits for the prophylaxis and therapy of metabolic disorders and for the encouragement of a salutary lifestyle [64].

## 6. Sleep Deprivation, Immune System and Viral Infections

Sleep and Immune System are closely and bidirectionally related. Several findings show that sleep deprivation is related to changes in the innate and adaptive immune system and is linked with various chronic diseases (such as diabetes, atherosclerosis and neurodegeneration), by promoting chronic, systemic low-grade inflammation [65,66,67]. Natural killer (NK) cell activity is altered after 40 h of waking state in humans and persistently declines after sleep deprivation with resumption of sleep [68]. The impact of sleep alteration and circadian imbalance on cortisol, inflammation mediators and cytokine microenvironment has also been reported [64].

Irwin MR et al. evaluated the effect of sleep alteration on the activation of inflammatory pathways. They found that in subjects partially sleep deprived, activation of physiological innate immunity and of STAT proteins occurred, maintaining inflammation beyond the natural threshold with possible consequences on the development of certain types of tumors [69].

According to Wright et al. [70], acute total sleep deprivation significantly increased serum cortisol levels, whereas chronic circadian misalignment significantly reduced cortisol levels. Circadian misalignment significantly increased plasma tumor necrosis factor-α (TNF-α), interleukin 10 (IL-10), interleukin 6 (IL-6) and C-reactive protein (CRP). Other authors confirmed that acute total sleep deprivation of 34 h duration induces secretion of pro-inflammatory cytokines, such as TNF-α, in healthy men. Prolonged sleep deprivation has been associated with increased serum IL-6 levels, both in humans [66,71] and animal study models [72].

Analysis of gene expression alterations with genome-wide microarrays has been performed in healthy controls subjected to sleep restriction. A considerable induction of genes involved in innate as well as in acquired immunity has been reported. B cell activation, interleukin-8 production, lipopolysaccharide binding are among the top up-regulated genes. As concerns T cells, up-regulation of some Th2 genes and downregulation of other genes involved in Th1 skewing suggest that sleep deprivation could also alter the balance between functional T lymphocytes [73]. In a recent study, authors analyzed with mass cytometry and single-cell RNA sequencing the characteristics of peripheral blood mononuclear cells obtained from healthy subjects sleep-deprived. They found expansion of T lymphocytes and plasma cells, increase of autoimmunity markers and altered subsets of myeloid cells, confirming the relationship between sleep alteration and immune dysregulation/chronic subclinical inflammation [74].

Lastly, total sleep deprivation has been reported to be related to enhanced daily tiredness, reduced efficiency, rise in pro-inflammatory cytokines and hormonal and metabolic disorders [75]. Moreover, population-based studies show that chronic sleep reduction increases mortality risk and promotes pathological conditions characterized by immune alterations and inflammation, including obesity, diabetes and cardiovascular diseases, MetS [76].

### 6.1. Sleep and Viral Infections

The outcomes of sleep deprivation on immune defense to influenza virus in the respiratory tract system have been reported in mice [77]. Mice after being orally immunized with influenza virus and nasally challenged, failed to mount an efficacious immune response to the virus when deprived of 7 h sleep compared to non-sleep deprived mice. Taken together, these data suggest that sleep is required for a functional immune system to face respiratory viral or bacterial infections [77]. Sleep also crucially affects the immune response in humans because sleep deprivation has been reported to decrease immune functions, favoring predisposition to viral infections. For instance, shorter sleep duration is associated with a higher risk for common cold [1]. Several explanations for the enhanced susceptibility to infections induced by reduced sleep have been proposed, comprising reduced T lymphocyte proliferation, reduced expression of HLA-DR molecules, increased CD14 positive cells and changes of CD4^+^ and CD8^+^ T lymphocytes, which have all been observed during partial sleep deprivation [1,70,75,77].

There is a growing consensus that the relationship between sleep disorders and viral infection may be bidirectional. Although viral infections can lead to a wide range of symptoms determined by the organ and system being infected, the majority of these manifestations tend to be accompanied by sleep disturbances in addition to fatigue and fever. In this regard, the effect of the influenza virus on sleep patterns has been probably the most extensively studied as a result of the fact that this virus can cross go through the blood brain interface and reach the CNS [1]. In particular, influenza virus intranasal inoculation in mice correlated with enhanced NREM and decreased REM, despite a decline in body temperature [78,79]. Intriguingly, the increase in NREM sleep during influenza appears to be strain-dependent as C57BL/6 (B6) mice experienced greater NREM sleep during infection, whereas BALB/c mice did not [80], suggesting that genetics may also influence the interplay between viral infection and sleep pattern [81]. Poliovirus is an additional pathogen that infects the CNS, leading to sleep alterations [82]. Patients experiencing an acute infection with poliovirus develop, several years later, a syndrome consisting of neuromuscular and respiratory symptoms and associated with sleep alterations, mainly characterized by obstructive apnea and hypopnea, with reduction of oxygen in blood during sleep [83]. Lastly, even asymptomatic patients infected by human immunodeficiency virus (HIV) experience fatigue and sleep [84]. In these patients, a significant decrease in REM sleep, total sleep time and slow-wave sleep (SWS) was followed by increased wakefulness [85]. These alterations could not be completely explained by patients’ underlying psychopathologies (e.g., anxiety disorders or depression) and side effects of medications (e.g., antiretrovirals), but they might also be caused by relationship between immune system and sleep [86]. To evaluate the role of the immune response in these alterations, the EEG changes in sleep patterns in infected immunosuppressed mice vs. immunocompetent mice were measured in one study [87]. As both groups experienced the same type of sleep pattern alterations, these might be directly caused by pathogenic processes driven by the virus instead of the immune response.

### 6.2. Sleep and SARS-CoV-2 Infection

Previous studies showed a bidirectional connection between sleep and viral infections [72,78,79,80,81,82,83,84] and growing evidence suggest that also the link between SARS-CoV-2 infection and sleep might be two-way as resumed in a recent review [88]. Several studies have recently highlighted the consistent role of SARS-CoV-2 infection on sleep characteristics, chronic fatigue, anxiety and depressive symptoms in caregivers, healthcare personnel, subjects affected by chronic diseases and healthy people due to extended lockdown [89,90,91]. A meta-analysis by Salari et al. summarizing the prevalence of anxiety, stress and depressive symptoms in the general population during pandemic reported prevalence rates of 31.9%, 29.6% and 33.7%, respectively [92]. On the other hand, two recent systematic meta-analysis using the Pittsburgh Sleep Quality Index (PSQI) estimated a prevalence of sleep problems respectively of 57% and 74.8% among COVID-19 patients compared to the general population (18% vs. 32.3% respectively) [89,93]. The study by Jahrami et al. [89] showed that male sex and older age in the COVID-19 subgroup were associated with higher rate of sleep disturbances. These findings are suggestive of a potential multifactorial, bi-directional relationship between sleep quality and SARS-CoV-2 infection. At the same time, an overlap between psychological distress, poor mental health and sleep problems was highlighted especially during the lockdown period [94].

As a result, an increase of mental illnesses in the Chinese population during the COVID-19 epidemic spread and a higher risk of displaying psychological issues in healthcare workers were reported [95].

To strengthen the bidirectional link between SARS-CoV-2 infection and sleep disturbances, an interesting study by Kim et al. conducted in six countries assessing sleep habits and a self-reported burnout among high-risk healthcare workers, showed that longer sleep duration was associated with lower odds to contract COVID-19 infection and conversely, having severe sleep problems was associated with 88% greater odds to contract COVID-19 infection. Moreover, the authors observed that daily burnout due to work was associated with 2.6-fold greater odds of COVID-19 infection with a greater duration and severity of the disease. They postulated that severe sleep problems, sleep deprivation and high levels of burnout may be a possible risk factor for COVID-19 infection especially among healthcare workers [96].

Among patients hospitalized for COVID-19, a recent observational comparative study assessing quality and quantity of sleep, confirmed that hospitalized COVID-19 patients were five times more likely to suffer from total sleep deprivation compared to non-covid patients [97]. It is still not clear if sleep disturbances can be promoted by the hospitalization in itself [98] or by an effect of symptoms such as anxiety or dyspnea [99] or if the penetration of the virus in the CNS (cerebrospinal fluid) may have an influence on them [100].

Moreover, bad sleep has been shown to impair the immune response, possibly leading to immunosuppression and facilitating the spread of infectious diseases and the worsening of mental health and quality of life [101]. A pivotal regulator of the sleep-immune system is Melatonin [102]. The circadian rhythm is strongly dependent on the release of this hormone with anti-inflammatory and immunomodulatory effects and the secretion of melatonin is synchronized with the rhythm of the immune system. For this reason, a disrupted circadian rhythm caused by poor sleep may decrease night-time melatonin levels increasing the susceptibility for SARS-CoV-2 infection [103]. Recent research showed a potential therapeutic role of melatonin and melatonergic drugs on K18-hACE2 mice infected with SARS-CoV-2 [104].

Moreover, sleep disturbances lead to an increase of pro-inflammatory cytokines receptors (IL-6 and TNF-alpha) and decrease of anti-inflammatory cytokines receptors (IL10) increasing the risk of infection [105].

A recent, interesting narrative review by Peters et al. commented on how neuroendocrine–immune mechanisms related to stress might take part in the spreading of SARS-CoV-2 infections and affect the development of COVID-19, pointing out that not every type of stress is harmful and that some stress could even reduce the risk and the progression of infection [106].

Evidence from the literature has shown that SARS-CoV-2 can frequently affect the nervous system and sleep impairment is the most frequent neurological symptom especially in the early phases of the infection [107].

A systematic review and meta-analysis of psychiatric, neuropsychiatric and sleep disorders associated with severe coronavirus infection highlighted that COVID-19 consequences may be multifactorial including the severity of infection, the immunological state, the social isolation, the treatments and hospitalization [108].

Several sleep disorders (e.g., sleep apnea) have been proposed as risk factors for severe COVID-19 [109,110]. Obstructive sleep apnea syndrome (OSAS) was found to be correlated with poor COVID-19 outcomes and a recent meta-analysis showed a correlation between OSAS and an enhanced risk for more severe disease, mortality, need for mechanical ventilation and ICU admission [111].

In a recent study conducted in the Chicago metropolitan area, Maas et al. showed that patients with OSAS experienced a risk of COVID-19 infection almost 8 times higher compared to people of the same age, treated in a large, multi-ethnic and socioeconomically different healthcare system. Among COVID-19 patients, OSAS was associated with a higher risk of hospital admission and approximately twofold risk for developing respiratory failure [112]. These findings may be explained by different mechanisms: repeated airway obstruction episodes in OSAS patients might impair sleep quality causing intermittent hypoxia and frequent awakenings, thus promoting lung inflammation [113] and exacerbating the cytochine storm related to ARDS [114], OSAS could also facilitate SARS-CoV-2 infection by a dysregulation of the renin-angiotensin system promoting an easier entrance of the virus in host cells [115], and once infection had occurred, increase the risk of cardiovascular conditions, such as arrhythmias, cardiac ischemia and a hypercoagulability state, leading to an unfavorable clinical progression [116]. In a systematic review including eighteen studies, risk factors for OSAS have been associated with poor COVID-19 outcomes [117]. Finally, considering that sleep disorders like insomnia may conduct to a compromised immunity and therefore to a sub-optimal vaccine-induced antibody response [118], a recent, interesting suggestion postulated the possibility for comorbid sleep-related breathing disorders, i.e., Comorbid Sleep Apnea (COMISA) to be an additional risk factor for reduced response to the COVID-19 vaccination [119]. Hence, it has been suggested that encouraging a better community sleep hygiene may be a tool to improve sleep quality in order to obtain a positive modulating effect in vaccine efficacy against COVID-19 [120].

## 7. Implications of the Findings and Future Perspectives

Sleep deprivation in rats strongly correlates with a peculiar condition characterized by progressive energy rise, specific lesions of the skin, alterations of the mechanisms of thermoregulation and final death [3]. A cause–effect link between sleep deprivation and immune suppression has also been proposed in humans, in whom poor sleep has been found to correlate with MetS symptoms. Treating sleep disorders by restoring adequate sleep time and quality has been shown to reduce glucose and energy metabolism abnormalities [56].

**Figure 1:**
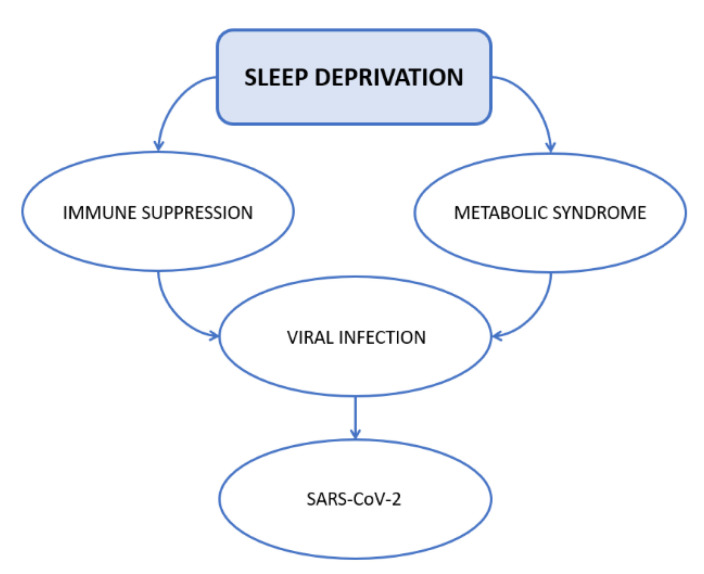
Possible mechanisms of interaction between sleep deprivation and increased risk of SARS-CoV-2 infection.

Being a hallmark of poverty, obesity correlates with limited access to high-quality health care. Given that some consequences of obesity, such as inflammation, might contribute to COVID-19 severity in individuals with high BMI [121], individuals sleep disorders and OSAS might facilitate SARS-CoV-2 infection. Obese COVID-19 patients affected by sleep disturbances might have a higher risk for cardiovascular conditions, leading to an unfavorable clinical progression [116] (Figure 1).

Recent literature data suggest that in addition to the well-established association between BMI-based obesity and severe COVID-19 outcome, the body fat distribution is also important; in particular, visceral adipose tissue and upper abdominal circumference have been proposed as simple tools for risk assessment in COVID-19 patients [122,123,124,125]. More recently, an interesting prospective multicenter study on hospitalized COVID-19 patients with respiratory failure showed that a larger neck circumference phenotype patient is more prone to have a negative outcome, a large neck being associated with an increased proinflammatory and prothrombotic status. Neck circumference in fact was found to be an independent predictor for mortality. The authors concluded that neck circumference may be a simple, additional tool providing additional prognostic information to BMI in hospitalized COVID-19 patients with respiratory failure [126].

## 8. Conclusions

In animal and human models, sleep deprivation correlates with immune suppression [7,13]. Obesity and diabetes have worryingly spread in industrialized countries. However, recognized risks factors like over-eating, nutritional choices of scarce quality and sedentary lifestyle cannot fully account for the increased prevalence of metabolic disease. Alteration of sleep and of the circadian rhythm have been identified as possible new risk factors for alteration of physiologic metabolism [64].

COVID-19 remains a serious public health concern worldwide as it has already affected millions of people. As obesity has been positively associated with infection severity [121], we strongly suggest screening for sleep disorders to enable a more comprehensive assessment of risk factors and development and implementation of more accurate prevention strategies and therapeutic interventions in particularly fragile individuals.

## Figures and Tables

**Table 1:** Major sleep deprivation effects in rats.

Symptoms	Effects
Mortality	Rats died or presented signs of imminent death usually after two or three weeks of TSD, unless sleep was recovered. PSD rats also died after 4–6 week.
Food intake	Despite increased food intake, with increased caloric values, TSD and PSD rats lost weight.
Appearance	Rats progressively appeared scrawny and debilitated.
Skin	Rats progressively appeared scrawny and debilitated.
Thermoregulatory changes	TSD rats showed an initial increase and subsequent decrease in waking T_ip_. PSD rats only showed T_ip_ decline.
Paradoxical sleep	Recovery from prolonged TSD determined large rebounds of PS. The same was demonstrated after only 2–4 days of TSD [3].

TSD: total sleep deprivation, PSD: paradoxical sleep deprivation, EE: energy expenditure, PS: paradoxical sleep, T_ip_: intraperitoneal temperature.

**Table 2:** Host defense effects in TSD rats.

Immune System Effects	Findings
Increased permeability of the gut wall	Gut wall of TSD rats becomes porous letting bacteria migrate to the peritoneal cavity [8].
Generalized failure of immune function	Skin lesions without inflammation and lack of fever [9]
Abnormal T lymphocyte-mediated response	TSD rats injected with allogenic tumor cells develop slow-growing tumors, which regress more rapidly than yoked controls. Time onset of the reaction is typical of a T mediated response [10].

TDS: total sleep deprivation.
